# Pulmonary immune responses to 2009 pandemic influenza A (H1N1) virus in mice

**DOI:** 10.1186/1471-2334-14-197

**Published:** 2014-04-12

**Authors:** Jin Lv, Dan Wang, Yan-Hong Hua, Shi-Jia Pei, Jin Wang, Wen-Wei Hu, Xi-Liang Wang, Na Jia, Qi-Sheng Jiang

**Affiliations:** 1 The Second Artillery General Hospital, PLA, Beijing 100088, China; 2 State Key Laboratory of Pathogen and Biosecurity, Beijing Institute of Microbiology and Epidemiology, 20 Dong-Da Street, Fengtai District, Beijing 100071, P.R. China; 3 Department of Biochemistry, the University of Hong Kong, Pokfulam, Hong Kong, China; 4 Cellular Technology Ltd, No.1 Qijie, Road Shangdi, Haidian District, Beijing 100085, China

**Keywords:** Pandemic influenza, Immunology, BALB/c mice

## Abstract

**Background:**

Well-characterized mice models will afford a cheaper, easy-handling opportunity for a more comprehensive understanding of 2009 influenza A (H1N1) virus’s pathogenesis potential. We aimed to provide a robust description of pulmonary immune responses in the mice infected by the virus.

**Methods:**

BALB/c mice were inoculated intranasally with A/Beijing/501/2009(H1N1) (BJ501) and A/PR/8/34(H1N1) (PR8) viruses and compared for survival rate, viral replication, and kinetics of pulmonary immune responses.

**Results:**

BJ501 virus replicated less efficiently in the lungs than PR8, and both caused lethal illness in the mice. The transient increases of pulmonary TNF-α 2 days post infection for BJ501 and of INF-γ and IL-10 at 6 days post infection for PR8 were observed. IL-2^+^ and IL-4^+^ secreting cells showed significant increase 12 days post infection, while IFN-γ^+^, IgG^+^ and IgA^+^ secreting cells increased 6 days post infection. The different patterns of pulmonary immunological parameters between two viruses were at most seen in IL-6, IL-17 secretion and IgG1/IgG2a ratio.

**Conclusions:**

The BALB/c mouse is evaluated as a good pathogenic model for studying BJ501 2009 H1N1 virus. The work provided some basic and detailed data, which might be referred when further evaluating innate and adapted pulmonary immune responses and local viral load in mice.

## Background

During March and early April 2009, a sharp increase in patients with influenza-like illness and associated hospitalizations and deaths in several areas of Mexico was notified, which was later proved to be caused by a novel swine origin 2009 influenza A (H1N1) virus (2009 H1N1) [1]. The virus was first identified in April from two unrelated pediatric febrile patients in southern California [2]. Genetic analysis demonstrated the 2009 H1N1 virus contained a unique combination of gene segments from both North American and Eurasian swine lineages that has not been identified previously in either swine or human populations [3]. By August 1, 2010, almost every country had reported laboratory confirmed cases, with over 18449 total death [4].

The main concerns about a new influenza pandemic are the disease burden and associated mortality it may cause. These are largely due to pneumonia resulting from viral infection. Many 2009 H1N1 infected patients have required hospitalization because of severe pneumonia and respiratory failure, with a fatal outcome [5]. Previous study indicated that 2009 H1N1 virus replicated efficiently in the mice lungs and resulted lesions in the lung histologically [6-8]. These suggest the mice model might be proper for 2009 H1N1 virus infection without mouse adaptation.

2009 H1N1 virus has many different properties on its replication and induced pulmonary immune responses in the lungs. 2009 H1N1 virus caused more severe pulmonary lesion than seasonal influenza viruses in animals [8,9]. In addition, the lower glycan binding properties also seemed to correlate with less-efficient transmission of 2009 H1N1 viruses in ferrets compared to seasonal H1N1 viruses [8]. In addition, the pathogenesis of lung lesions caused by 2009 H1N1 viruses might be different from 1918 pandemic virus and highly pathogenic avian influenza H5N1 virus, as no major “cytokine storm” was observed with 2009 H1N1 infection of the human cell lines [10,11] and the mice lungs [6]. This lower cytokine production of 2009 H1N1 virus infection compared to 1918 virus and H5N1 virus might be associated with the findings that the virus did not spread to extrapulmonary organs and caused no viremia in ferrets or mice [8,9]. Also, 2009 H1N1 virus differs in modest but subtle ways from seasonal influenza virus for cytokine dysregulation *in vitro* and in *ex vivo* cultures of human cell lines [10-12].

Thus, we want to study 2009 H1N1 virus pulmonary infection in the mice to provide a raw data on the Th1/Th2 cellular immune responses and IgG, IgA, IgM, and IgG subtype antibodies humoral immune responses at different days post infection.

## Methods

### Viruses

A virulent strain of influenza A virus, A/PR/8/34(H1N1) (PR8), was kindly provided by Dr. Yuelong-Shu (Chinese Center for Disease Control and Prevention). Influenza H1N1 A/Beijing/501/2009(BJ501) virus was isolated and obtained from our institution. Virus stocks were propagated consistent with the original isolate passage in the allantoic cavity of 10-day-old embryonated hens’ eggs. Each egg was injected with 0.1 ml normal saline containing 1 haemagglutinin unit (HAU) of virus, incubated for 48 h at 34°C, and then held at 4°C overnight. The allantoic fluids were harvested, pooled, centrifuged to remove cellular debris and stored at −70°C until use. Virus stocks were low-passage-number viruses (E1-E2). For some assays, virus stock was further purified by centrifugation through a discontinuous sucrose gradient at 30,000 × *g* for 3 h at 4°C. The milky virus band located in the upper half of the gradient was harvested, washed in phosphate-buffered saline (PBS), and centrifuged at 24,000 × *g* for 4 h at 4°C. The virus pellet was gradually re-suspended in PBS, and aliquots were stored at −70°C. Virus stocks were quantified by a haemagglutination assay to obtain HAU. TCID_50_ was determined by titration in Madin-Darby canine kidney (MDCK) cells, with daily observation of cytopathic effect, and confirmed by haemagglutination assays. Viral titers were titrated by standard plaque assays in MDCK cells as previous described for determination of PFU titers [13]. MDCK cells routinely maintained in Dulbecco’s Modified Eagle’s medium plus 10% fetal bovine serum (FBS). The 50% egg infectious dose (EID_50_) was determined by serial titration of virus stock in eggs, and EID_50_/ml values were calculated according to the method of Reed and Muench [14]. All animal experiments with 2009 H1N1 viruses were conducted under biosafety level 3 enhanced (BSL3+) containment in accordance with guidelines of the World Health Organization [15].

### Infection of mice

Female BALB/c mice (Institute of Jingfeng Medical Laboratory, Beijing, China), 6 to 8 weeks of age, were anesthetized with diethyl ether and inoculated intranasally (i.n.) with 25 μl of infectious virus diluted in sterile phosphate-buffered saline (PBS). Fifty-percent lethal dose (LD_50_) titers were determined by the inoculating groups of 6 mice i.n. with serial 10-fold dilutions of virus. LD_50_ titers were calculated by the method of Reed and Muench [14]. For determination of lung virus titers, morbidity (measured by weight loss), and mortality at multiple time points during the two weeks of infection, 30 additional mice were infected i.n. with inoculating dose (5 × 10^5^, 5 × 10^4^ and 5 × 10^3^ p.f.u.) of virus. Individual body weights from six mice were recorded for each group on days 0, 2, 4, 6, 8, 10, 12 and 14 post infections (p.i.) and monitored daily for disease signs and death for 14 days p.i. On days 2, 4, 6, 8, 10, 12 and 14 p.i., fifteen mice with inoculating dose (5 × 10^5^ p.f.u.) of virus at each time point were euthanatized, lungs were collected separately and homogenized in 2 mL sterile PBS. After this process, homogenates were frozen separately in sterile tubes at −80°C for later titration of virus loads, antibody detection and cytokine determination. For the detection of lung immune cells and ELISPOT assay, eight to ten mice at each time point post infection were sacrificed by cervical dislocation. Single-cell suspensions of the lungs were prepared as follows. Freshly resected lungs were placed into cold DMEM, grinded gently and the suspensions were pelleted, resuspended and passed through nylon mesh filters. Red blood cells were removed by lysis buffer treatment (BD biosciences). Cells were counted and resuspended at appropriate concentrations for each particular experiment. The animal experiments were approved by the Animal Subjects Research Review Board of the Beijing Institute of Microbiology and Epidemiology and were conducted according to the institution's guidelines for animal husbandry.

### Real-time reverse-transcriptase-polymerase-chain-reaction (RT-PCR) detection of the viral load

The viral load in the lungs was determined by real time RT-PCR. RNA was extracted from the lungs by homogenizing with Trizol (Invitrogen, USA) reagent. Then, reverse transcription was performed by targeting a conserved region of the influenza matrix (M) gene for PR8 as previously described [16]. For the detection of BJ501, the primers and probe were designed according to the CDC protocol for swine influenza A virus [17].

### Flow cytometry

The lungs were harvested at various time points post infection. Single cell suspensions were stained with fluorochrome-labeled anti-CD3, anti-CD4, anti-CD8, anti-CD11b, and anti-CD11 cantibodies (BD Biosciences). Cells were labeled for 45 min at 4°C in staining buffer (PBS with 1% FBS, 0.02% NaN3) and then washed twice with PBS, and fixed overnight at 4°C with 2% paraformaldehyde. Flow cytometry was performed on a FACS Aria flow cytometer (BD Biosciences). The frequency of neutrophils (CD11b+/CD11c-/Ly6G/c+) was determined by appropriate gating within the total lung leukocytes. For determination of frequency of CD4^+^T cells and CD8^+^T cells, the CD3^+^T cells were first determined by appropriated gating within the pulmonary lymphocytes, and then CD4^+^/CD8^−^T cells and CD4^−^/CD8^+^T cells were detected by gating within the CD3^+^T cells.

### ELISPOT (enzyme-linked immunospot) assay for Cytokine Secreting Cells (CSCs)

An ELISPOT assay was conducted as previously described [18]. Briefly, ELISPOT 96-well plates (Millipore) were coated with anti-mouse IFN-γ, IL-2, and IL-4 capture antibody and incubated for 24 h at 4°C (BD Biosciences). The following day, the plates were washed and blocked with 200 μL of 1640 medium containing 10% fetal calf serum for 2 hours at room temperature to decrease the number of remaining protein-binding sites. Two hundred thousand cells of lung cell suspensions were added to each well and stimulated overnight at 37°C in 5% CO2 in the presence of RPMI 1640 (negative control), Con A (positive control), purified PR8 or BJ501 antigens (2.5 μg/ml). After 24 h of stimulation, the cells were washed and incubated for 24 h at 4°C with biotinylated anti-mouse IFN-γ, IL-2 and IL-4 antibodies (BD Biosciences). The plates were washed, and streptavidin-alkaline phosphatase (BD Biosciences) was added to each well and incubated for 2 h at room temperature. The plate was washed, 100 μl of AEC solution (BD Biosciences) were added to each well. The plate was then rinsed with distilled water and dried at room temperature. Spots were counted by an automated ELISPOT reader (CTL Limited).

### ELISPOT assay for Antibody Secreting Cells (ASCs)

The numbers of influenza-virus-specific Antibody-Secreting Cells were determined using the ELISPOT assay, as described previously [19]. Briefly, a 96-well plate with a nitrocellulose base (Millititer HA; Millipore) was coated with 2.5 μg/ml purified PR8 or BJ501 antigen, as an irrelevant-antigen control, wells were coated with phosphate-buffered saline containing 7.5 μg/mL BSA, then blocked with 200 μL of 1640 medium containing 10% fetal calf serum. A total of 5 × 10^6^/ml cell suspensions from the lungs were added to each well and the mixture incubated for 9 h. The plate was washed with PBS–Tween and incubated with goat anti-mouse IgA (eBionsciences) or goat anti-mouse IgG (BD Biosciences) conjugated with biotin. Then, streptavidine conjugated with alkaline phosphatase (BD Biosciences) was added to each well and the plates were incubated for 1 hour at room temperature. The plates were washed as before with PBS-T and PBS and 100 μl/well of AEC solution were added. The reaction was stopped after 10–30 minutes at room temperature in dark by thorough rinsing with a tap water. Spots were counted by an automated ELISPOT reader (CTL Limited).

### Antibody assays

Influenza-specific pulmonary IgA, IgM and IgG isotypes antibodies were measured by enzyme-linked immunosorbent assay (ELISA), using plates coated with 1 μg/ml per well of purified PR8 or BJ501 influenza virus. The reactions were detected by goat anti-mouse IgA, IgM (Invitrogen), goat anti-mouse IgG1, IgG2b, IgG2a, IgG2c, and IgG3 (Bethyl laboratories, Inc.), goat anti-mouse IgG (Jackson ImmunoResearch Laboratories) antibodies conjugated to horseradish peroxidase. The plates were developed with TMB (Sigma, USA). Absorbance was read at 450 nm on a Bio-RAD model 550 Microplate Reader. The virus-specific Ab titer was defined as the reciprocal of the highest sample dilution giving an absorbance value greater than twice that of the samples from the negative controls. The titers were gained in duplicate.

### Cytokine measurement

The lungs were homogenized on ice individually in 2 ml PBS. Cytokine protein levels were measured by a specific ELISA assay following the kit description (R&D Systems, Minneapolis, MN, USA). The detection limits for the ELISA development kits are as follows: IFN-γ, 15-10000 pg/mL; TNF-α, 15-10000 pg/mL; IL-6: 15-10000 pg/mL; IL-17: 15-10000 pg/ml; IL-10, 15-2000 pg/mL.

### Statistical analysis

Statistical differences at each time point within one group were determined by one-way ANOVA test, while statistical differences of the two viruses at the same time-point were determined by independent-sample T Test with SPSS19.0. Values of *p*<0.05 were considered significant.

## Results

### Comparisons of survival rate, weight loss and viral load in the mice

**Figure 1:**
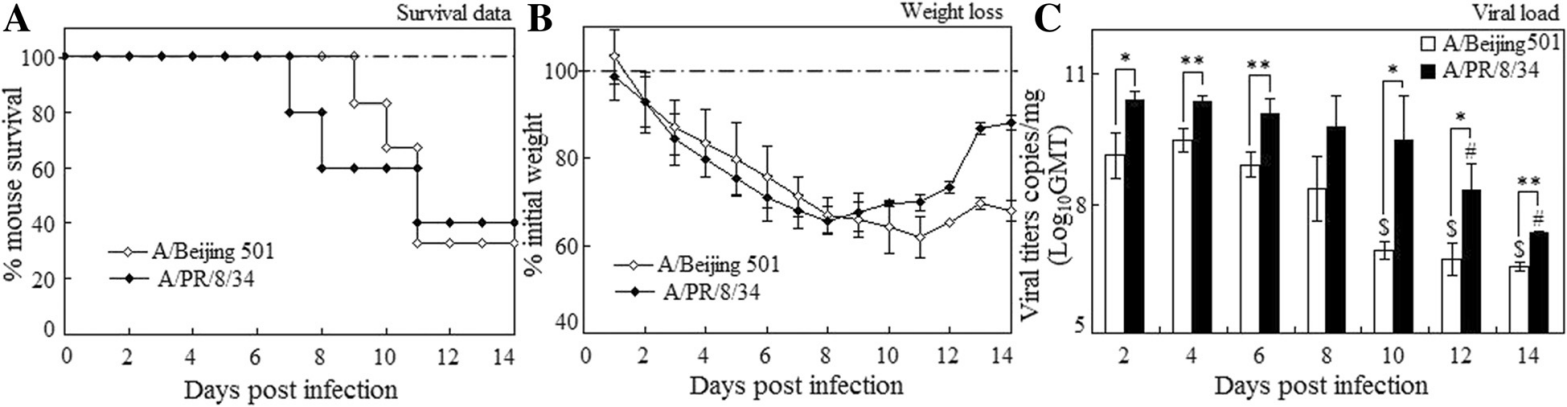
**Virulence of two strains of influenza A virus on mice.** BALB/c mice were intranasally infected with 5 × 10^5^ p.f.u. dose of A/Beijing/501/2009 (H1N1) and A/PR/8/34 (H1N1) viruses. **(A)** Survival data and **(B)** Weight loss **(C)** Viral growth in the lung at different time points. Viral loads were determined in homogenates prepared from lungs at certain time points post-infection by real time RT-PCR test, and were expressed as viral RNA copies per miligram lung tissue. Mean viral load is representative of 5–6 mice lungs per time point group, and I bars indicated the standard deviations. The dotted lines were the control level. $ *p* < 0.05, compared among different time-points within BJ501 viruses infection, # compared among different time-points within PR8 viruses infection, *p < 0.05 and **p < 0.01, compared between BJ501 and PR8 infections at the same time-point.

In this study, we used the same infectious doses of two influenza viruses, although the LD_50_ was different between PR8 viruses and BJ501 viruses (the LD_50_ of PR8 was 5.0 × 10^3^ p.f.u/25 μL while BJ501 was 7.76 × 10^3^ p.f.u/25 μL respectively). Three doses (5 × 10^5^, 5 × 10^4^ and 5 × 10^3^ p.f.u. i.n.) were initially used and the lower groups (5 × 10^3^, 5 × 10^4^) showed a significant higher survival rate in BJ501 infections than in PR8 ones (data not shown). No significant differences of survival rate and weight lose were observed in high (5 × 10^5^) dose infection (Figure 1A, B), and this dose was used in the following infections. The decrease of viral load was significantly earlier in the lungs of mice infected with BJ501 viruses (from day 10 post infection) than that in the PR8 infection (from day 12 post infection). On the other hand, pulmonary viral load was significantly higher in PR8 infection than in BJ501 group (Figure 1C) (*p* < 0.05).

### The kinetics of pulmonary immune cell responses and cytokine productions in the mice

**Figure 2:**
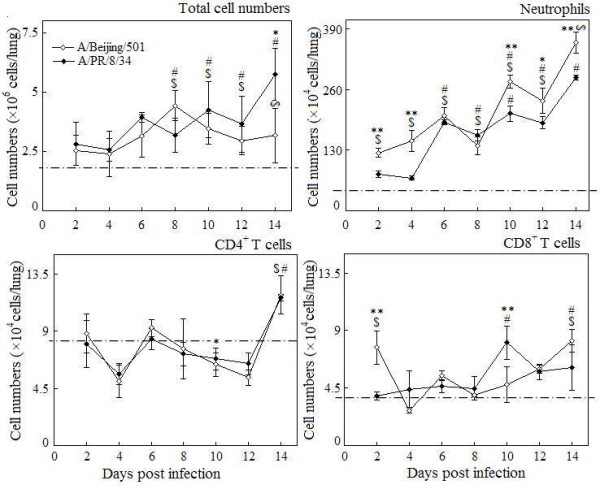
**The kinetics of pulmonary immune cells responses induced by influenza virus infection.** Mice without infection were used as the control group. Mean cell numbers were representative of 3 mice lungs per time point group, and I bars indicated the standard deviations. The dotted lines were the control level. $ *p* < 0.05, compared among different time-points within BJ501 viruses infection, # compared among different time-points within PR8 viruses infection, *p < 0.05 and **p < 0.01, compared between BJ501 and PR8 infections at the same time-point.

**Figure 3:**
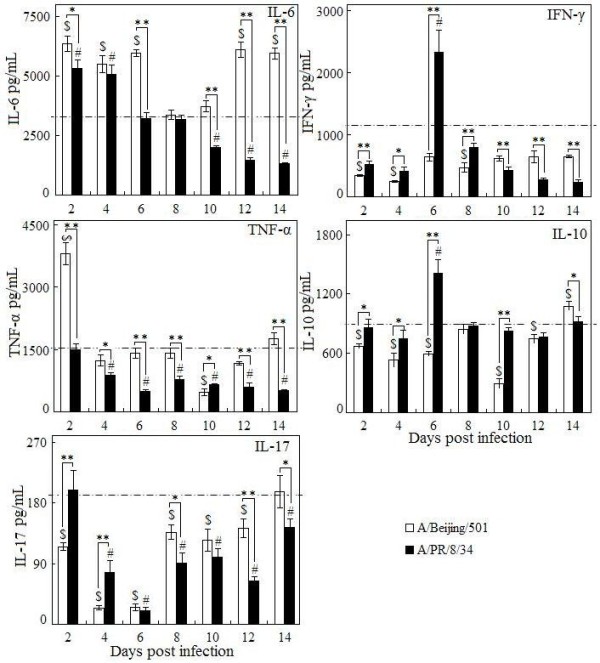
**Determination of cytokine levels in the lungs of mice per time-point with influenza virus infection.** Five to six mice were euthanized at each time point post infection, and lung homogenates were generated for analysis. IL-6, TNF-α, IFN-γ, IL-10, and IL-17 levels were assayed by ELISA as described in Methods. Mice without infection were used as the control group. Columns represented mean cytokine concentrations representative of 5–6 mice, and I bars indicated the standard deviations. The dotted lines were the control level. $ *p* < 0.05, compared among different time-points within BJ501 viruses infection, # compared among different time-points within PR8 viruses infection, *p < 0.05 and **p < 0.01, compared between BJ501 and PR8 infections at the same time-point.

As expected, total pulmonary cells accumulated 6 days post infection in local in both virus infections (Figure 2). The neutrophils significantly increased as early as 2 days post infection with BJ501, and this increasing was 4 days earlier than that of PR8 infection (Figure 2). The increase of CD4^+^T cells was only observed in the late infection with both viruses (14 days p.i.) (Figure 2). A transient increase of CD8^+^T cells at 2 days post infection with BJ501 was observed, but CD8^+^T cell decreased to normal level until days 14 (Figure 2). Pulmonary cytokine excretions were seen in Figure 3. In the BJ501 infection group, TNF-α increased significantly on days 2 but dropped to control level immediately in the following days. IL-6 increased from days 2 to days 6 and from days 12 to days 14 post infection. Both IFN-γ and IL-10 did not significantly change during the whole infection. However, IL-17 significantly decreased from 4 to 6 days and then recovered to control level on days 8 post infection (Figure 3). Otherwise, in the PR8 infection group, IFN-γ, and IL-10 transiently increased at days 6 post infection, and then both decreased to control level in the late stage of infections. The earliest cytokine noted was IL-6, which increased at 2 days, then dropped to control level at 6 days post infection. IL-17 however, showed significantly decreased level during the infection, especially after 2 days post infection (Figure 3).

### Antibody responses in the lung of mice

**Figure 4:**
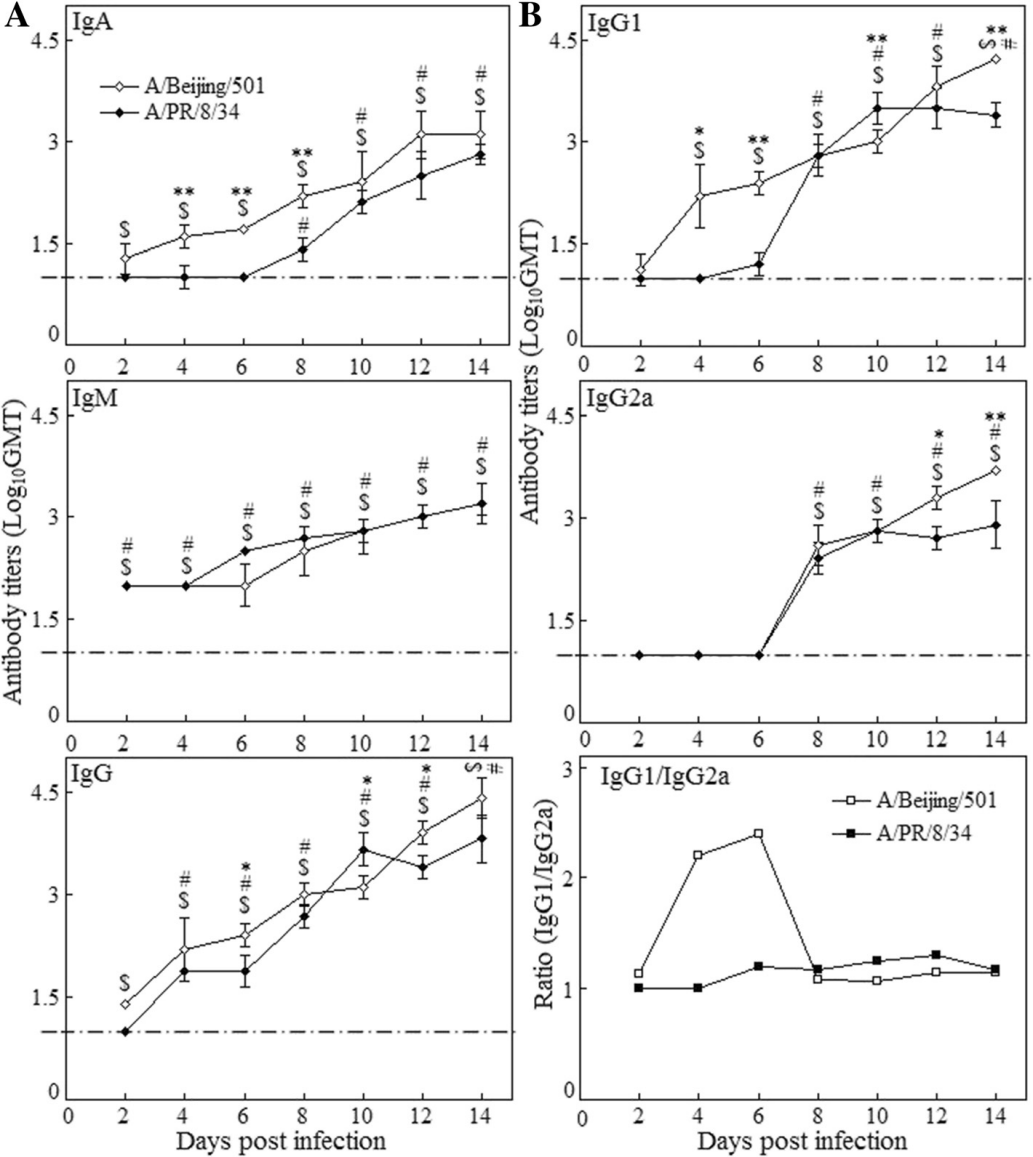
**Kinetics of antibody responses in the lungs of mice with influenza virus infection.** Kinetics of IgA, IgM, IgG, **(A)** and IgG2a, IgG1 antibody responses and IgG1/IgG2a ratio **(B)** in the lungs of mice after 5 × 10^5^ p.f.u. A/Beijing/501/2009(H1N1) and A/PR/8/34/(H1N1) viruses infection. Mice were sacrificed at certain time points post infection, and virus-specific antibodies in the lungs were determined by ELISA. Mice without infection were used as the control group. I bars represent standard deviations. The dotted lines were the control level. $ *p* < 0.05, compared among different time-points within BJ501 viruses infection, # compared among different time-points within PR8 viruses infection, *p < 0.05 and **p < 0.01, compared between BJ501 and PR8 infections at the same time-point.

Figure 4A illustrated that IgA, IgM, and IgG antibodies all increased significantly during the infections. The isotype analysis showed IgG isotype had a similar dynamic pattern between two groups. Both IgG1, IgG2b, IgG2a and IgG3 subpopulation increased simultaneously, and IgG1and IgG2b were predominant. The level of IgG3 antibody titers was similar to that of IgG2a. No detectable IgG2c was found in this study (data not shown). In BJ501 infection group, the increase of IgG1 was 4 days earlier than that of IgG2a (Figure 4B). On days 14, the titers of IgG1 and IgG2a were 1:16218 and 1:5012 respectively. In PR8 group, both IgG1 and IgG2a increased significantly on days 8, and kept at high level till 14 days post infection, and the titers were 1:2512 and 1:794 respectively (Figure 4B). Data in Figure 4B illustrated that the ratio of IgG1 to IgG2a in the BJ501 group was higher than that in the PR8 group in the early infection, which suggested a stronger Th2 response predominance in the BJ501 infection.

### Influenza virus-specific CSCs and ASCs responses in the lungs of mice

**Figure 5:**
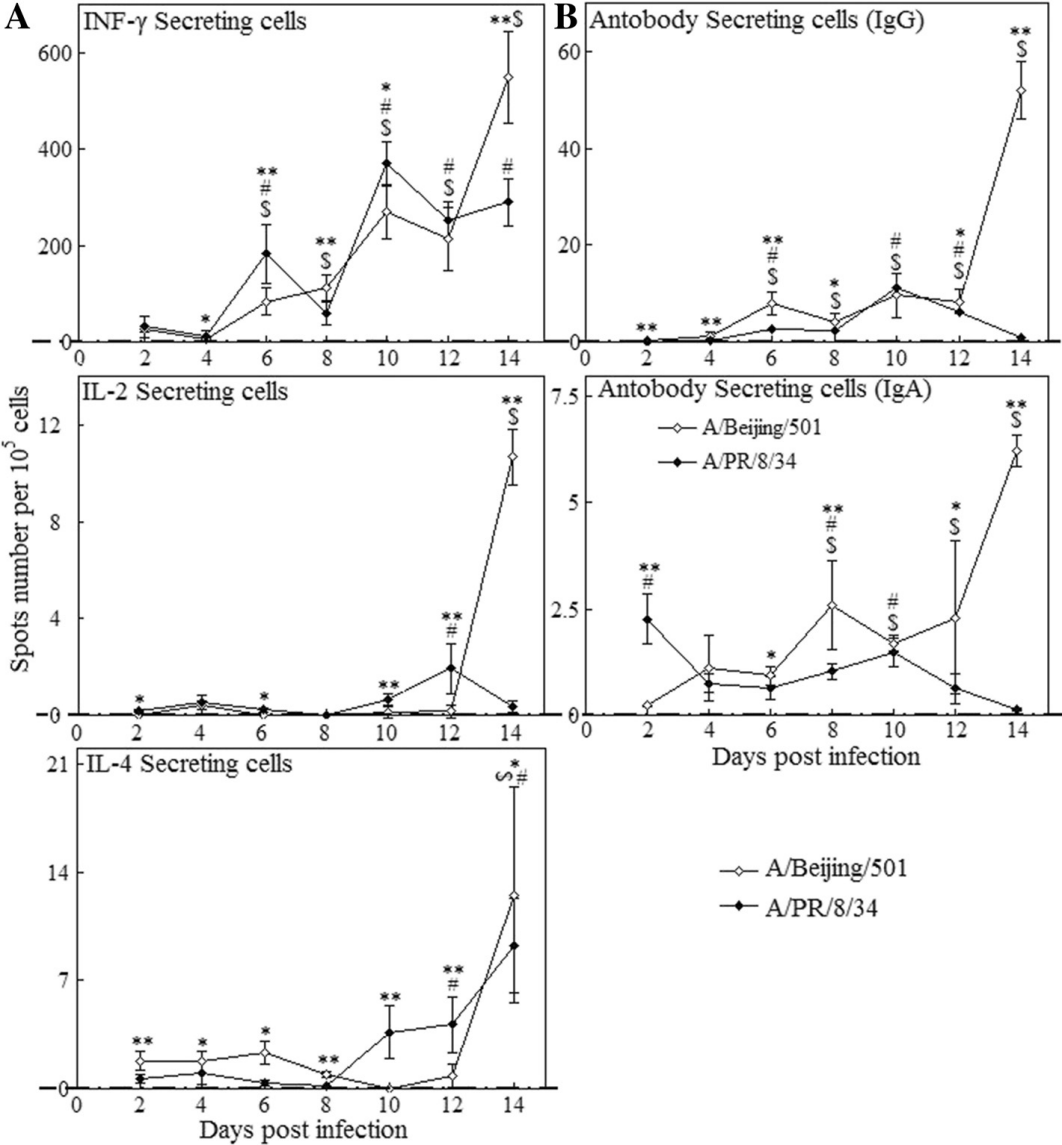
**Influenza virus-specific CSCs and ASCs responses in the lungs of mice with influenza viruses infection.** Influenza virus-specific cytokine secreting cells (CSCs) **(A)** and antibody secreting cells (ASCs) **(B)** responses in the lungs of mice with 5 × 10^5^ p.f.u. A/Beijing/501/2009(H1N1) and A/PR/8/34/(H1N1) viruses infection. Quantitative analysis of influenza-specific CSCs (IFN-γ^+^, IL-4^+^ and IL-2^+^) and ASCs (IgG^+^ and IgA^+^) was done with ELISPOT assay. I bars represent standard deviations. The dotted lines were the control level. $ *p* < 0.05, compared among different time-points within BJ501 viruses infection, # compared among different time-points within PR8 viruses infection, *p < 0.05 and **p < 0.01, compared between BJ501 and PR8 infections at the same time-point.

The cytokines (IFN-γ^+^, IL-2^+^, IL-4^+^) secreting cells (CSC) and antibody (IgG^+^ and IgA^+^) secreting cells (ASCs) to influenza virus antigens in the lungs of mice were investigated by the ELISPOT assay. We found that IFN-γ^+^ CSC response was highest and its pattern was similar between two viruses, although by the end of observation IFN-γ^+^ CSC response in the BJ501 were much higher than that in the PR8 infection (548.54 and 290.23 CSCs per 10^5^ cells respectively). The response level and peak day of IL-2^+^ and IL-4^+^ CSCs induced by the BJ501 showed a little difference from those by the PR8 (Figure 5A). Figure 5B showed that IgG^+^ ASCs to influenza BJ501 antigens was similar with that to PR8 from days 6 to days 12 post infection, but on days 14, BJ501 group showed significant higher response than PR8 one (Figure 5B). IgA^+^ ASCs in the BJ501 group increased significantly on days 8, then dropped slightly on days 10 but was still significant higher than that in the control and then kept increasing from days 12 to days 14 post infection. In contrast, the level of IgA^+^ ASCs in the PR8 group increased significantly in the early stage of infection, then dropped to control level immediately from days 4 to days 8, and on days 10 it increased a little, and kept at control level from days 12 to 14 (Figure 5B).

## Discussion

To evaluate effective therapies and elucidate influenza pathogenesis, it is essential to have laboratory animal models that replicated the major features of illness in human. The animal models currently in use for influenza include mice, ferrets, rats [20], pigs [21], cats [22], dogs [23], and nonhuman primates [24]. Mice and ferrets are currently the most intensively employed models, and mice remain the primary model due to their cost, inbreeding, and ability to utilize genetically modified animals. Influenza-virus-infected ferrets develop many of the typical signs of infection in humans [25]. The limitations of the ferret model are the lack of specific reagents in studying immune system and the availability of animals without prior exposure to influenza [26]. Our study demonstrated that 2009 H1N1 virus replicated efficiently in the lung of mice, and for severe infections and for lethal mice experiment as in this study, a high viral load might be present, and 2009 H1N1 virus seemed to induce a distinct pattern of cytokine markers associated with the pathogenesis.

The most outstanding cytokine is IL-6. Its correlation with severe infections of 2009 H1N1 virus was demonstrated in both human cases [27,28] and in high dose inoculated mice model [7]. In our mice experiment, we found IL-6 significantly increased not only in the early but also in the late stage of infection. IL-6, a multifunctional cytokine expressed by both lymphoid and non-lymphoid cells [29], played a central role in resolving the innate immune response and directing the transition from innate to adaptive immunity [30], and IL-6 concentrations were significantly correlated with the symptoms and signs of influenza A infection in human [31,32], whereas depletion of IL-6 did not directly protect against death from lethal H5N1 influenza virus infection [33]. Recently, IL-6 is found to be in the regulation of Th17 differentiation [34]. Th17 cell also secrets cytokine of IL-17, which is critical in the immunopathology of acute lung injury due to acute influenza infection [35].

However, the *in vivo* role of IL-17 in the clearance of influenza virus and its immune pathology has been controversial. Several studies have demonstrated the important protective role of IL-17 against lethal influenza infection. In contrast, other groups have showed that IL-17 signaling was not required for viral clearance, but rather played a pathogenic role by recruiting neutrophils to the site of inflammation [27,36-38]. Furthermore, IL-17 deficiency or treatment with monoclonal antibodies against IL-17-ameliorated acute lung injury induced by the 2009 H1N1 virus in the mice [39]. In our mice experiment, we found significant decreased pulmonary IL-17 both in BI501 and PR8 infection, whether this correlated with the virus replication in local deserved further study.

TNF-α is an important proinflammatory cytokine located at the downstream of IL-17. Previous study found it also had a positive feedback effect on Th17 *in vitro*[40]. The transient increase of TNF-α at days 2 might contribute in the enhancement of chemokine gene expression, macrophages and dendritic cells maturation, and further activation of antigen presentation [41]. TNF-α level has been found to correlate directly with the severity of gross and histologic lung lesions in the influenza infected mice [42]. Daniela Damjanovic and his colleagues suggested that TNF-α was dispensable for influenza clearance. Their study showed that TNF-α was up-regulated in the lung after influenza infection and TNF-α deficiency led not only to a greater extent of illness but also to heightened lung immunopathology and tissue remodeling [43]. Taken together, proinflammatory cytokine (TNF-α) and mediators involved in the development of Th17 (IL-17, IL-6) responses were associated with the critical illness of 2009 pandemic influenza infection either in the detrimental or beneficial role. The IL-6 pathway had been a promising new therapeutic target in treatment of autoimmune illness [44,45]. The use of anti-inflammatory and immunomodulatory agents for pandemic influenza control should be largely evaluated in case of antiviral resistance [46].

Several studies had indicated that the increased pathogenicity of influenza A virus may be associated with extremely rapid recruitment of macrophages and neutrophils into the lungs in fatal infection [47,48]. In addition, in the late recovery phase, macrophage and neutrophils may be in primary cooperation with the antibody working at the late-phase clearance of influenza virus through phagocytic elimination of infected cells [49,50]. Contrast to IFN-γ^+^ CSCs increasing from the early infection, IL-2^+^ and IL-4^+^ CSCs only increased 12 days post infection, which implied the strongly stimulated adapted immune response. In addition, we found that BJ501 virus also induced relatively stronger Th2 mediated responses (pulmonary IgA^+^ and IgG^+^ secreting cells, IgG2/IgG2a ratio) in the lung than PR8 virus. These stronger Th2 immune responses might be correlated with the relatively less efficient virus replication in the lung.

The analysis of known molecular determinants that confer pathogenicity and antiviral drug resistance in 2009 H1N1 viruses was previously discussed [3]. Viruses with lysine at position 627 replicated better at 33°C, the temperature found in the human upper respiratory tract [51]. The mutations (HA, T197A; NA, V106I and N248D; PA, P224S; NP, V100I) between BJ501 and Cal04 were also found in A/Mexico/4482/09(H1N1) strain, which was isolated from a fatal case [6]. The HA 227 substitution from alanine in PR8 to glutamic acid in BJ501 and Cal04 was located in the glycan receptor-binding sites and antigenic loops [52]. All these mutations (see Table 1) should be further characterized of their functional determinants.

**Table 1:** Amino acid differences among BJ 501, Cal 04 and PR 8 influenza viruses

**Gene**	**Mutation**	**Effect**	Strains*			**Reference**
			**BJ 501**	**Cal 04**	**PR8**	
PB2	E627K	−627 K associated with increased transmission, advantageous replication at 33°C and confers air transmission of avian virus.	E	E	K	[53]
		-Important determination of host range. Avian strains have 627E, human strains 627 K.				
		-627 K is linked to increased virulence in mice.				
PA	S224P	Unknown	S	P	S	
	P275L	Unknown	L	L	P	
NS1	P42S	−42S is critical for virulence of H5N1 strains and to antagonize host cell interferon induction	S	S	S	
HA	A227E	Unknown	E	E	A	
	P83S	Unknown	S	P	P	
	A197T	Unknown	A	T	A	
	I321V	Unknown	V	I	I	
NP	D16G	Unknown	G	G	D	
	V100I	Unknown	I	V	V	
NA	H274Y	-Oseltamivir resistance is associated to H274Y	Y	Y	S	
	I106V	Unknown	I	V	I	
	D248N	Unknown	D	N	D	
M2	S31N	-Confers cross-resistance to adamantane drugs	N	N	N	
M1	L42F	Unknown	F	L	L	
	D94N	Unknown	N	D	D	

*BJ501, A/Beijing/501/2009(H1N1); Cal04, A/California/04/2009(H1N1); PR8, A/PR/8/34(H1N1).

## Conclusions

Although the global wave of 2009 H1N1 virus has went away, many unsolved questions are still important to be further clarified for prevention and control of potential pandemic threat. This study provided some basic data on the optimal time to observe different immune parameters, for example, IL-2^+^, IL-4^+^ CSCs stimulated about 12 days post infection, TNF-α, IFN-γ, IL-10 cytokines only transiently increased in the early infection. It is hoped this paper will contribute for immunological researchers to the better use of mice experiment to study the correlation between the viral load and critical immune response in the lung.

## Competing interests

The authors declare that this work does not involve competing interests.

## Authors’ contributions

JL, DW, XLW, QSJ and NJ conceived and designed the study. Experiments were performed by JL, YHH, and WWH. Data were analysed by JL, SJP, JW and NJ. All authors read and approved the final manuscript.

## Pre-publication history

The pre-publication history for this paper can be accessed here:

http://www.biomedcentral.com/1471-2334/14/197/prepub
